# Guanine nucleotide exchange factor 2 for Rab5 proteins coordinated with GLUP6/GEF regulates the intracellular transport of the proglutelin from the Golgi apparatus to the protein storage vacuole in rice endosperm

**DOI:** 10.1093/jxb/erv325

**Published:** 2015-07-01

**Authors:** Liuying Wen, Masako Fukuda, Mariko Sunada, Sonoko Ishino, Yoshizumi Ishino, Thomas W. Okita, Masahiro Ogawa, Takashi Ueda, Toshihiro Kumamaru

**Affiliations:** ^1^Faculty of Agriculture, Kyushu University, Fukuoka 812–8581, Japan; ^2^Tobacco Research Institute, Chinese Academy of Agricultural Science, Qingdao 266101, China; ^3^Graduated School of Science, University of Tokyo, Tokyo 113-0033, Japan; ^4^Institute of Biological Chemistry, Washington State University, Pullman, Washington 99164-6340, USA; ^5^Department of General Education, Yamaguchi Prefectural University, Yamaguchi 753–8502, Japan; ^6^Japan Science and Technology Agency (JST), PRESTO, Saitama 332-0012, Japan

**Keywords:** Guanine nucleotide exchange factor, helical bundle domain, intracellular transport, *in vitro* GEF assay, Rab5, storage protein.

## Abstract

Rice glutelin polypeptides are initially synthesized on the endoplasmic reticulum (ER) membrane as a proglutelin, which are then transported to the protein storage vacuole (PSV) via the Golgi apparatus. Rab5 and its cognate activator guanine nucleotide exchange factor (GEF) are essential for the intracellular transport of proglutelin from the Golgi apparatus to the PSV. Results from previous studies showed that the double recessive type of *glup4/rab5a* and *glup6/gef* mutant accumulated much higher amounts of proglutelin than either parent line. The present study demonstrates that the double recessive type of *glup4/rab5a* and *glup6/gef* mutant showed not only elevated proglutelin levels and much larger paramural bodies but also reduced the number and size of PSVs, indicating a synergistic mutation effect. These observations led us to the hypothesis that other isoforms of Rab5 and GEF also participate in the intracellular transport of rice glutelin. A database search identified a novel guanine nucleotide exchange factor, Rab5-GEF2. Like GLUP6/GEF, Rab5-GEF2 was capable of activating Rab5a and two other Rab5 isoforms in *in vitro* GTP/GDP exchange assays. GEF proteins consist of the helical bundle (HB) domain at the N-terminus, Vps9 domain, and a C-terminal region. By the deletion analysis of GEFs, the HB domain was found essential for the activation of Rab5 proteins.

## Highlight

Both GLUP6/GEF and the novel Rab5-GEF activate multiple Rab5 isoforms which participate in the intracellular transport of proglutelin from the Golgi apparatus to the protein storage vacuole.

## Introduction

Rice seed storage proteins consist of three types: glutelin, prolamine, and α-globulin, which are soluble in acid and/or alkali, alcohol and salt solutions, respectively. During seed development, glutelin polypeptides are initially synthesized on the endoplasmic reticulum (ER) membrane as a proglutelin (Yamagata *et al.*, 1982). This precursor form is then transported to the protein storage vacuole (PSV) via dense vesicles formed from the Golgi apparatus (Krishnan *et al.*, 1986; Yamagata and Tanaka, 1986), where it is cleaved into acidic and basic subunits. These proteins together with the α-globulins accumulate to form the protein body (PB) type II (Yamagata *et al.*, 1982).

In order to elucidate the mechanism of intracellular transport of the proglutelin from the ER to PSV, we selected and identified mutants, *endosperm storage protein2* (*esp2*) and *glutelin precursor* (*glup*) 1–7 (Kumamaru *et al.*, 2007; Ueda *et al.*, 2010) that accumulated abnormal amounts of proglutelin in developing rice endosperm. The *glup4* and the *glup6* mutants among of them were identified as loss-of-function (null) mutations of the small GTPase Rab5a and its cognate activator, guanine nucleotide exchange factor (GEF), respectively (Fukuda *et al.*, 2011, 2013). Homologous mutations for Rab5a (*gpa1*: OsRab5A) and GEF (*gpa2*: OsVPS9A) were isolated and characterized by the Wan laboratory (Liu *et al.*, 2013; Wang *et al.*, 2010).

GLUP6/GEF was demonstrated to activate GLUP4/Rab5a as well as *Arabidopsis* Rab5 isoforms (Fukuda *et al.*, 2013). GLUP4/Rab5a and GLUP6/GEF are required not only for the intracellular transport of proglutelins from the Golgi to PSV in rice endosperm but also in the maintenance of the general structural organization of the endomembrane system in developing rice seeds (Fukuda *et al.*, 2011). The endosperms in *glup4* and *glup6* mutants exhibit similar defective phenotypes with elevated proglutelin levels and the novel appearance of paramural bodies (PMBs), with subcellular structures initially formed by abortive endosomal uptake of secreted glutelins, α-globulins and Golgi and post-Golgi components, and subsequently by direct secretion of these constituents into this novel cellular structure (Fukuda *et al.*, 2013).

Rab GTPases are involved in various membrane trafficking events including endosome organization, cytokinesis, trafficking from the Golgi to the plasma membrane and to vacuoles (Woollard and Moore, 2008). The Rab5 members in plant are located not only on the late endosomal compartment, the prevacuolar compartment (PVC), but can also be found on the Golgi and the early endosomal trans-Golgi network as well (Sohn *et al.*, 2003; Bolte *et al.*, 2004; Kotzer *et al.*, 2004; Lee *et al.*, 2004; Fukuda *et al.*, 2011; Haas *et al.*, 2007; Stierhof and El Kasmi, 2010). In *Arabidopsis*, three Rab5 orthologues, RHA1/RABF2a, ARA7/RABF2b and ARA6/RABF1, have been identified. ARA7 and RHA1 are conventional Rab5 types, while ARA6 is a plant-specific type conserved in land plants (Ueda *et al.*, 2001; Ebine *et al.*, 2011). Both the conventional type Rab5s, Ara7 and Rha1, and plant-specific Rab5 and Ara6, participate in endosomal trafficking to the vacuole and/or the plasma membrane (Ueda *et al.*, 2001, 2004; Sohn *et al.*, 2003; Kotzer *et al.*, 2004; Lee *et al.*, 2004; Ebine *et al.*, 2011). Although all three Rab5s are located on the multivesicular body/PVC, plant-specific and conventional types are localized on different populations of membrane-bounded compartments in a partially overlapping manner (Ueda *et al.*, 2004; Haas *et al.*, 2007).

Several GEFs for Rab5, have been reported including the yeast Vps9p, *Arabidopsis* VPS9a and various animal forms such as Rabex-5, RIN-1, RIN-2, RIN-3, RME-6, Alsin and ALS2CL (Carney *et al.*, 2006; Goh *et al.*, 2007). They all share a highly conserved VPS9 domain (Burd *et al.*, 1996; Horiuchi *et al.*, 1997). The *Arabidopsis* VPS9a serves as a common activator for the two plant Rab5 types (ARA7, RHA1, and ARA6) (Goh *et al.*, 2007). Mutations in *VPS9a* result in embryonic lethality and various developmental defects including abnormal root development. Moreover, abnormally enlarged PMBs containing membrane vesicles and accumulation of the 12S globulin precursor were observed in *vps9a* mutant (Goh *et al.*, 2007; Ebine *et al.*, 2011). These facts suggest that VPS9a is essential for endosomal trafficking and development in *Arabidopsis*.

In our previous study, analysis of F_2_ seeds from a cross between *glup4* and *glup6* lines showed a portion of seeds containing proglutelin levels significantly higher than either parent line (Ueda *et al.*, 2010). This result suggested that pyramiding of both recessive genes mediated a synergistic elevation of proglutelin levels. Rice has several Rab5 homologues expressed during seed development (Fukuda *et al.*, 2011; Liu *et al.*, 2013). Based on these lines of evidence, we hypothesized that the synergistic effect seen with the combined *glup4* and *glup6* mutations reflected the existence of other Rab5(s) and/or GEF(s) participating in intracellular transport of rice glutelin. In this study the double recessive type of *glup4/rab5a* and *glup6/gef* mutation displays much more severe phenotypic alterations in protein trafficking than either parent alone. Moreover, we present evidence that other Rab5 and GEF homologues participate in the trafficking of proglutelin from the Golgi to the PSV.

## Materials and methods

### Plant materials

The *glup6* line EM939 and *glup4* lines EM425 and EM956, which were induced by *N*-methyl-*N*-nitrosourea (MNU) mutagenesis and which accumulated elevated amounts of proglutelin (Satoh-Cruz *et al.*, 2010b; Ueda *et al.*, 2010), were used in these experiments. The double recessive type of *glup4* and *glup6* mutant line was isolated from the progenies of the cross between EM939 and EM425 or between EM939 and EM956. All F_2_ seeds obtained from self-pollinated F_1_ plants were bisected into embryo and non-embryo portions. The proteins extracted from the non-embryo portion of individual F_2_ seeds were analysed by SDS-PAGE. Those containing substantially higher levels of proglutelin than either parent line were identified, with the embryo portion of the seed sown in soil and F_2_ plants cultivated to maturity. By sequencing genomic DNA from the F_2_ plants, the double recessive type for both genes was confirmed. Developing seeds, 1 to 3 weeks after flowering (WAF), of the double recessive type were analysed as described below.

### SDS-PAGE and western blot analysis

Extraction of the proteins from seeds, SDS-PAGE, and western blot analysis was performed as described previously (Ushijima *et al.*, 2011; Fukuda *et al.*, 2013).

### DNA sequence analysis

DNA sequencing analysis was performed as described previously (Kumamaru *et al.*, 2010). The sequence of total genomic DNAs, extracted by the CTAB (cetyltrimethylammonium bromide) method (Murray and Thompson, 1980) from the leaves of the double recessive type, was determined using an ABI PRISM 3100 Genetic Analyzer (Applied Biosystems Ltd.). DNA sequence analysis was performed using EditView1.0.1 and AutoAssembler 2.1. Comparisons between the double recessive type, two single mutants, and wild type were performed using CLUSTALW of DNA Data Bank of Japan (DDBJ: http://clustalw.ddbj.nig.ac.jp/index.php?lang=en).

### Microscopic analysis

For immunofluorescence and immunoelectron microscopy studies, the samples were fixed in LR white resin and analysed as described previously (Takemoto *et al.*, 2002). For transmission electron microscopy, samples were embedded in epoxy resin and analysed as described previously (Nagamine *et al.*, 2011).

### Antibodies

Antibodies against glutelin and α-globulin were raised in mice and rabbits, respectively (Fukuda *et al.*, 2013). Antibodies against the 14kDa prolamine were raised in rabbits (Nagamine *et al.*, 2011).

### Identification and classification of novel GEFs containing VPS9 domain

To detect novel GEF isoforms of GLUP6/GEF, the SALADA database was searched for proteins containing the Vps9 domain. Genes encoding for proteins containing the conserved Vps9 domain were annotated by RAP-DB and expression confirmed by RiceXpro. The deduced amino acid sequences corresponding to the GEF genes were aligned using ClustalW of DDBJ. A phylogenetic tree was constructed using the neighbor-joining method as implemented in the MEGA5.0 programme according to the results of ClustalW analysis (Supplementary Fig. S5).

### Expression and purification of GST fusion proteins

GST-conjugates of GLUP6/GEF, Rab5-GEF2, GLUP4/Rab5a, Rab5b, Rab5c, Rab11, and truncated proteins of GEFs were produced in *Escherichia coli* BL21 (DE3) using the pGEX 4T-1 expression vector (GE Healthcare) and purified according to the procedure described earlier (Goh *et al.*, 2007). The primer sequences for construction of the expression plasmids are shown in Supplementary Table S1 and the experimental procedures are described in the table legend. All Rab proteins were purified in the GDP-bound form in the presence of Mg^2+^ and without EDTA or GDP. After purification of GST-tagged proteins by chromatography on a Glutathione-Sepharose 4B column (GE Healthcare), the purified proteins were loaded onto a desalting column (GE healthcare) to remove glutathione and then stored frozen at −80ºC. The purification procedure was completed in one day to maximize enzyme activity.

### Guanine nucleotide exchange assay

The guanine nucleotide exchange assay was performed according to the method described previously (Goh *et al.*, 2007). Each purified GST-Rab protein was preloaded with a 25M excess of GDP at 25ºC for 2h. The excess GDP was removed by a desalting column. For each assay, 1 µM GST-Rab GDP form was pre-incubated with or without GST-GEFs in GEF assay buffer containing 20mM Tris-HCl, pH 8.0, 150mM NaCl, and 0.5mM MgCl_2_ for 100 s. The nucleotide exchange reaction was started by the addition of 0.1mM GMP-PNP. Trp intrinsic fluorescence of Rab proteins was detected at 340nm by the excitation at 298nm using a fluorescence spectrophotometer (F-2500, Hitachi). Each experiment was completed within two days as Rab GTPase activity decreased rapidly after thawing.

### Surface plasmon resonance analysis

Surface plasmon resonance was performed using a BIACORE J system (GE Healthcare UK Ltd.) to analyse protein-to-protein interactions. To monitor the interactions of GLUP4/Rab5a with Rab5-GEF2 and GLUP6/GEF, highly purified GLUP4/Rab5a protein was immobilized on a Sensor Chip CM5 according to the manufacturer’s protocol. Various concentrations of purified Rab5-GEF2 and GLUP6/GEF were then applied to the GLUP4/Rab5a immobilized Sensor Chips for 120 s in 10mM HEPES-NaOH, pH 7.4, 0.15M NaCl, 0.5mM MgCl_2_, 10mM GDP, and 0.1% Triton X-100 at a flow rate of 30 μl/min. The apparent equilibrium constants (*K*_D_) for GLUP4/Rab5a with Rab5-GEF2 and GLUP6/GEF were calculated from the association and dissociation curves using the BIA evaluation software (GE Healthcare UK Ltd.).

### Accession numbers

Sequence data from this article can be found in the GenBank/EMBL data libraries under the following accession numbers: GLUP4/Rab5a (AK061116, Os12g0631100), Rab5b (AK121527, Os03g0151900), Rab5c (AK067459, Os03g0666500), Rab11 (AK103220, Os08g0525000), GLUP6/GEF (AK070551, Os03g02 62900), Rab5-GEF2 (LOC_Os03g62580.1), ΔC-terminal Rab5-GEF2 (AK070821).

## Results

### The double recessive type of *glup4/rab5a* and *glup6/gef* mutant accumulates more proglutelin than each single mutant type

We had previously reported that a subset of F_2_ seeds from a cross between *glup4* and *glup6* lines showed a more pronounced abnormal accumulation of proglutelin than seeds from the two parents alone (Ueda *et al.*, 2010). In order to determine whether the *glup4* and *glup6* mutations interacted synergistically to further elevate proglutelin accumulation, plant lines containing both recessive genes were identified by sequencing the *GLUP4/Rab5a* and *GLUP6/GEF* genes in F_2_ plants obtained from a cross between *glup4/rab5a* lines, EM425 or EM956, with the *glup6/gef* line, EM939.

**Fig. 1.**
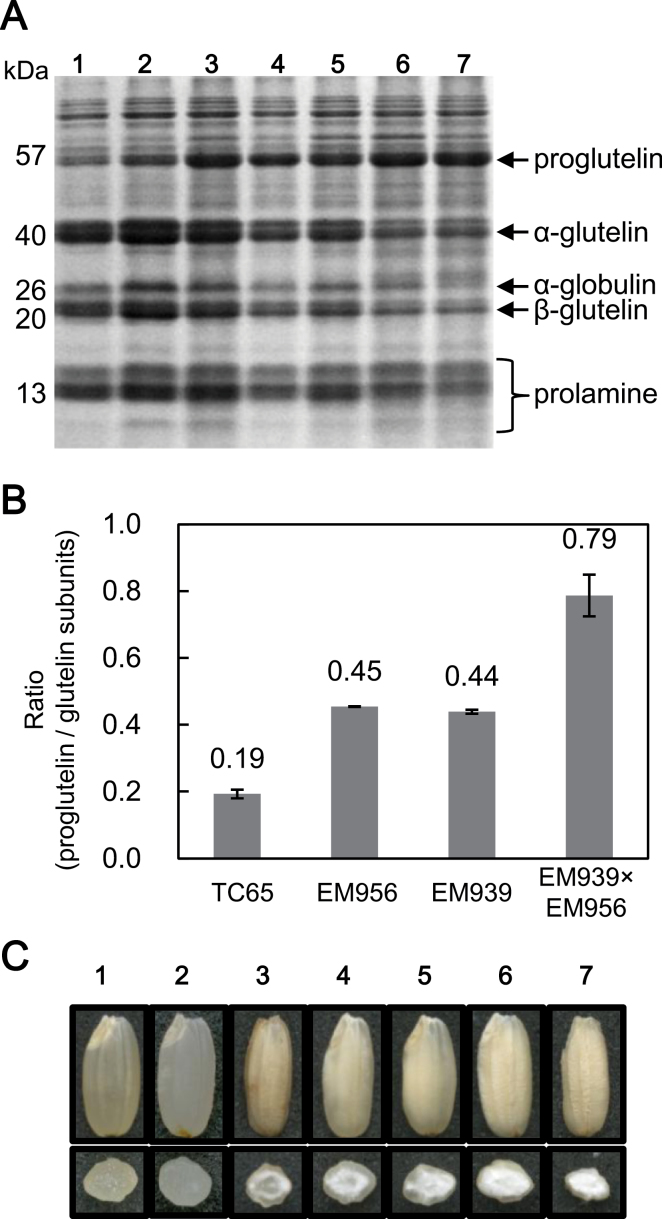
SDS-PAGE of seed storage proteins in the double recessive type of *glup4/rab5a* and *glup6/gef*. (A) CBB staining of the gel. (B) Ratio of proglutelin to sum of glutelin subunits. This value was calculated from densitometry of image A. (C) The floury endosperm of mutant lines. Lane 1, the wild type, Taichung65; lane 2, the wild type, Kinmaze; lane 3, *glup4/rab5a*, EM425; lane 4, *glup4/rab5a*, EM956; lane 5, *glup6/gef*, EM939; lane 6, double recessive type from the crossing of EM425 and EM939; lane 7, double recessive type from the crossing of EM939 and EM956.

The self-pollinated seeds of the double recessive type were then analysed by SDS-PAGE (Fig. 1A). The levels of proglutelin and glutelin subunits in the double recessive type were substantially elevated and reduced, respectively, compared with each parent line (Fig. 1A). This view is readily evident by analysis of the ratio of proglutelin to glutelin subunits where the double recessive type has a much higher ratio than that seen for each parent line (Fig. 1B). In addition, the grain morphology of the double recessive type exhibited a more severe floury grain phenotype than each parent line (Fig 1C). These results suggest that the *glup4* and *glup6* mutations interact synergistically to elevate proglutelin and, in turn, reduce glutelin subunits as well as mediate a more severe chalky grain phenotype.

### Protein body formation is more severely disrupted in double recessive type of *glup4/rab5a* and *glup6/gef*

**Fig. 2.**
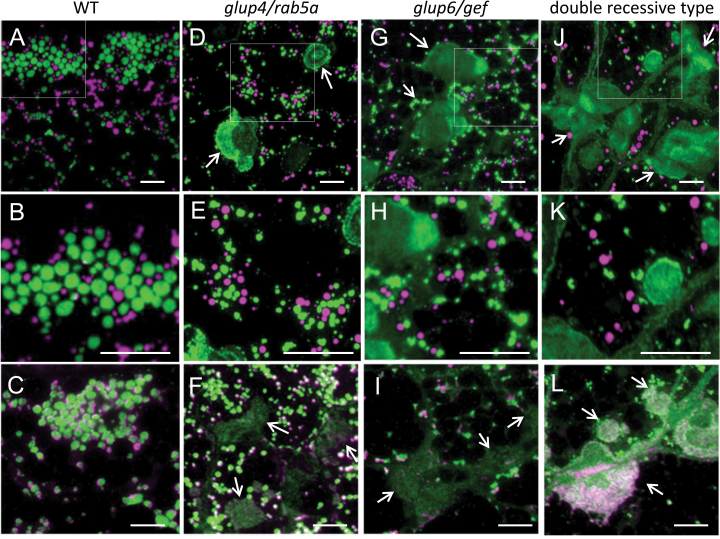
Immunofluorescence microscopy of the endosperm from the double recessive type of *glup4/rab5a* and *glup6/gef* at 2 WAF. A−C, wild type; D−F, *glup4/rab5a*; G to I, *glup6/gef*; J−L, double recessive type from the crossing of EM425 and EM939. B, E, H, and K are the enlarged images of the square areas in A, D, G, and J, respectively. A, B, D, E, G, H, J, and K: secondary antibodies labelled with rhodamine (magenta) and fluorescein isothiocyanate (FITC, green) were used to visualize the reaction of prolamine and glutelin antibodies, respectively. C, F, I, and L: secondary antibodies labelled with rhodamine (magenta) and fluorescein isothiocyanate (FITC, green) were used to visualize the reaction of α-globulin and glutelin antibodies, respectively. Arrows indicates the paramural bodies (PMBs). Bars, 10 μm.

In order to elucidate the influence of the combined *glup4/rab5a* and *glup6/gef* mutations on intracellular transport of proglutelins, protein bodies in the endosperm of the single and double recessive type were analysed by immunofluorescence microscopy (Fig. 2, Supplementary Fig. S1). In the wild-type endosperm, protein bodies containing prolamines (protein body type I: PB-I) and those containing glutelins and α-globulins (protein storage vacuole: PSV) increased in size and number as the seed develops (Fig. 2A−C, Supplementary Fig. S1A−C). In each mutant type, prolamine containing PB-I were estimated to be similar in size and number to that seen for the wild type (Fig. 2B, E, H, K). In contrast, glutelin-containing PSVs were smaller and fewer in number in each mutant type (Fig. 2E, H, K) compared with the wild type (Fig. 2B). The severity of this condition varied among the mutant types, with *glup4/rab5a* showing a moderate reduction in size and number of PSVs (Fig. 2D, E) followed by *glup6/gef* (Fig. 2G, H) while the double recessive type showed the largest reduction in number of PSVs (Fig. 2J, K). Nearly all of the synthesized α-globulin was packaged in paramural bodies (PMBs) in the double recessive type (Fig. 2L).

As described previously (Fukuda *et al.*, 2011, 2013), PMBs were conspicuous in each single mutant (Fig. 2D, G). Many cells contained multiple PMBs and these novel organelles were clustered at the cell’s periphery adjacent to the cell wall. This cell wall relationship is readily evident in the double recessive type as the extracellular space separating individual cells was swollen with glutelin and α-globulin storage proteins (Fig. 2J, K, L).

To examine the origin of PMBs in the double mutant, immunofluorescence studies were carried out with sections taken from developing endosperms of the wild type, each single mutant and double recessive type at 1 and 3 weeks after flowering (WAF) (Supplementary Fig. S1). At 1 WAF, glutelin-containing PSVs were beginning to be accumulated in the wild type, *glup4* and *glup6* (Supplementary Fig. S1A, D, G), whereas few PSVs were observed in the double recessive type with nearly all of the glutelins secreted and located at the cell’s boundary or in small PMBs (Supplementary Fig. S1J). This pattern was maintained at 3 WAF where few glutelin-containing PSVs were seen in the double recessive type with nearly all of the glutelins as well as α-globulins either secreted extracellularly or located in the PMBs (Supplementary Fig. S1K, L). Hence, normal transport and packaging of glutelins and α-globulins to PSVs were severely disrupted in the double recessive type than each single mutant.

**Fig. 3.**
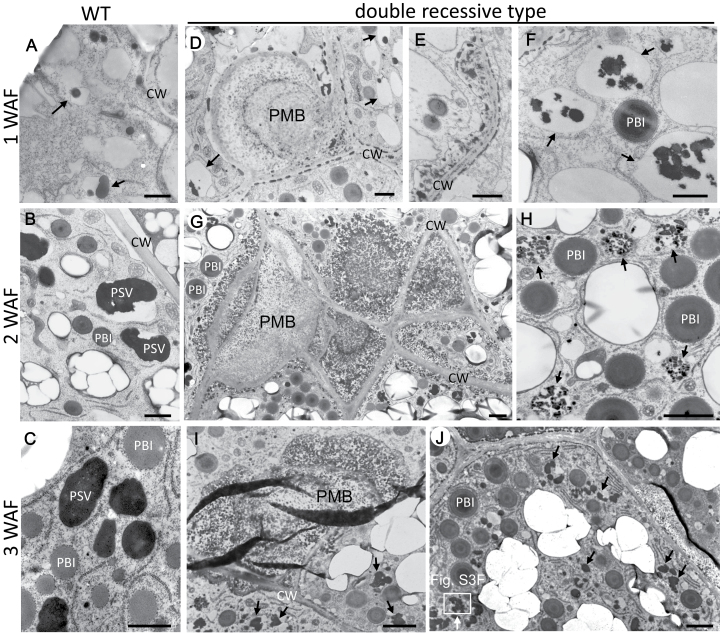
Transmission electron microscopy of the endosperm from the double recessive type of *glup4/rab5a* and *glup6/gef*. A−C, wild type; D−J, the double recessive type from the crossing of EM425 and EM939. A−C depict sections of developing wild-type endosperm at 1 WAF, 2 WAF and 3 WAF, respectively. Corresponding sections from the double recessive type are shown in D−F, G and H, and I and J. I, J: gold particles of 15nm and 5nm indicate the reaction of glutelin and prolamine antibodies, respectively. The square area in J shows the PSV is indicated as enlarged image in Fig. S3F. Arrows in A, D, F, H, I, and J show the PSVs. CW, cell wall; PMB, paramural body. Bars:1 μm in A to F; 2 μm in G to J.

**Fig. 4.**
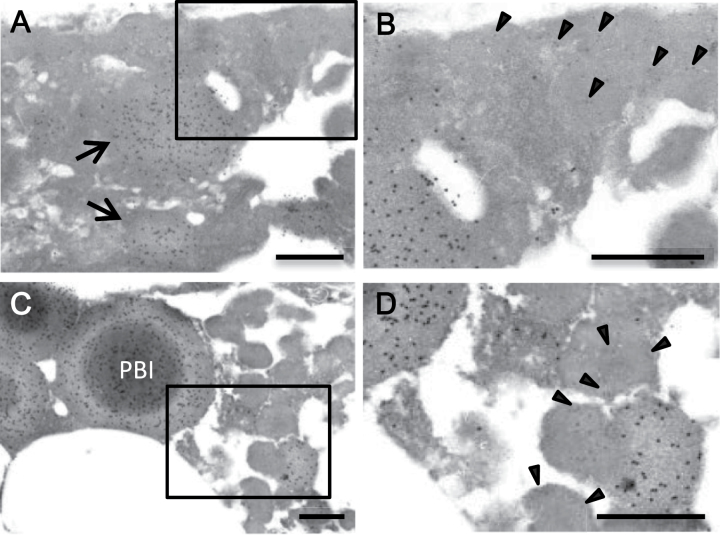
Electron dense inclusion of the endosperm from the double recessive type of *glup4/rab5a* and *glup6/gef* at 3 WAF. A−D: the double recessive type from the crossing of EM939 and EM956. B and D are the enlarged images of the square areas in A and C, respectively. Gold particles of 15nm and 5nm indicate the reaction of prolamine and glutelin antibodies, respectively. The arrows show the electron-dense material contained spherical granules of prolamine. The arrowheads in B and D show the gold particles by the reaction of glutelin antibodies. Bars, 500nm.

To obtain additional insight on the trafficking pathways of glutelin and α-globulin to the PSVs and PMBs in the double recessive type, transmission electron microscopy studies were conducted (Figs 3, 4; Supplementary Fig. S2). At 1 WAF, electron-dense granules and the PMBs were detected in the paramural space created by the invagination of the plasma membrane away from the cell wall in the double recessive type (Fig. 3D, E) as well as in each single mutant (Supplementary Fig. S2B, F) but not in the wild type (Fig. 3A). The extracellular electron-dense granules in the double recessive type were labelled with glutelin antibodies (Supplementary Fig. S3B). This observation together with the reduction of glutelin-containing PSVs in the cytoplasm indicates that proglutelin granules, likely in the form of Golgi-derived dense vesicles, were secreted (Fig. 3D). Some proglutelins were normally transported to PSV in the double recessive type (Fig. 3D, F) as well as in each single mutant (Supplementary Fig. S2A, E). In 2 WAF endosperm, the PMBs in the double recessive type were substantially larger than those observed at 1 WAF (Fig. 3G) and from those present in each single mutant (Supplementary Fig. S2C, G). Unlike the condition seen at 1 WAF, electron-dense granules were not readily observed in the cell wall areas suggesting that these granules were taken up by the PMBs. Similar to that seen for the cell wall-associated electron dense granules, those located in the PMB were labelled with glutelin antibody in the double recessive type (Supplementary Fig. S3D, E).

PSVs in *glup4* and *glup6* endosperms contained a single inclusion similar to those observed in the wild type. Some of the inclusions observed in the single mutant differ from the smaller and spherical type seen in the wild type in having an irregular shape (Fig. 3B, C; Supplementary Fig. S2D, H). By contrast, the PSVs of the double recessive type at 2 WAF were only partially filled and contained multiple small electron-dense granules (Fig. 3H). These small electron-dense granules, labelled with glutelin antibodies (Supplementary Fig. S3C) completely filled up the small irregularly-shaped PSVs of <1 μm at 3 WAF in the double recessive type (Fig. 3I, J arrows; Supplementary Fig. S3F).

Although many of the endosperm cells of the double recessive type contained normal PB-I and aberrant PSVs, some cells located close to the aleurone layer showed a large mass of electron-dense material in the cytoplasm at 3 WAF (Fig. 4). Immunocytochemical analysis showed that this electron-dense material contained spherical granules of prolamine (Fig. 4A arrows) embedded within the larger electron-dense matrix, which was labelled with glutelin antibodies (Fig. 4B arrowheads). Moreover, fusion of PB-I and PSVs was observed (Fig. 4C, D). These results indicate that in some cells of the double recessive type, the membrane fusion occurs among PB-I and PSVs.

The more severe biochemical and cellular structural changes observed in the double recessive type compared with the individual parent lines indicate that combining the *glup4/rab5a* and *glup6/gef* genes further disrupts the intracellular transport of proglutelins and α-globulins and the development of the protein bodies. The more severe effect seen when the *glup4/rab5a* and *glup6/gef* were combined led us to the hypothesis that homologues of GLUP4/Rab5a are also activated by GLUP6/GEF, and conversely that GLUP4/Rab5a and other Rab5 homologues are activated by other GEF proteins. Collectively, these redundant activities participate in the intracellular transport of the proglutelin from Golgi apparatus to the protein storage vacuole. To substantiate this hypothesis, Rab5 and GEF homologues were identified and their properties examined as described below.

### GLUP6/GEF activates other Rab5 members

Rice contains four Rab5 GTPases which fall into two subgroups: conventional Rab5 [GLUP4/Rab5a (Os12g0631100) and Rab5c (Os03g0666500)] and plant-specific Rab5 [Rab5b (Os03g0151900) and Rab5d (Os10g0441800)] (Fukuda *et al.*, 2011; Liu *et al.*, 2013). Analysis of the rice expression profile database (RiceXRro: http://ricexpro.dna.affrc.go.jp/) indicates that all four Rab5 GTPase genes are expressed in developing rice endosperm (Supplementary Fig. S4).

**Fig. 5.**
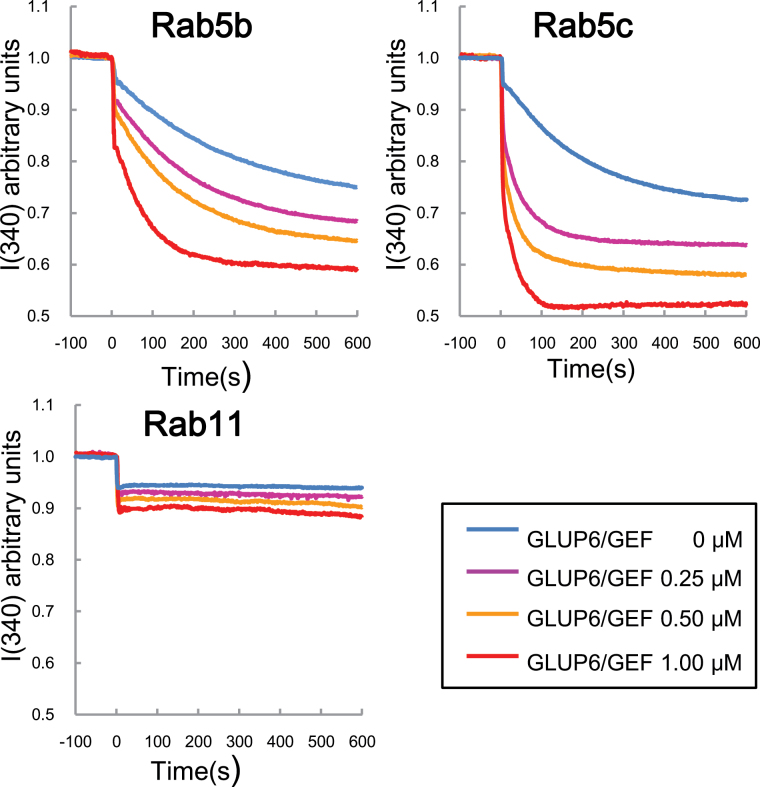
*in vitro* GEF assay of GLUP6/GEF. The conformational changes of Rab5s and Rab11 (as 1 μM GST fusion proteins) upon GDP/GMP-PNP exchange were measured by monitoring Trp autofluorescence when incubated with 0 μM (blue), 0.25 μM (magenta), 0.5 μM (yellow), and 1 μM (red) GST-GLUP6/GEF. Note that GST-GLUP6/GEF activates Rab5b and Rab5c, but not Rab11.

In order to elucidate whether the GLUP6/GEF can activate other Rab5s in rice, *in vitro* guanine nucleotide exchange assays using tryptophan autofluorescence were conducted using GST-tagged recombinant proteins as described previously (Fukuda *et al.*, 2013). In this study, Rab5b and Rab5c were selected as representative of the plant-specific type and conventional type of Rab5 isoforms, respectively. As shown in Fig. 5, Rab5b and Rab5c were activated by GLUP6/GEF in a dose-dependent manner, whereas, in contrast, Rab11 was not activated by GLUP6/GEF. These results indicate that GLUP6/GEF activates not only GLUP4/Rab5a but also at least two other Rab5 isoforms present in developing rice endosperm. Evidence for the genetic and physical interactions of GLUP6/GEF to Rab5 family proteins was also reported by Liu *et al.* (2013). Overall, the collective evidence suggests that, in addition to GLUP4/Rab5a, other Rab5 isoforms such as Rab5b and Rab5c may participate in glutelin trafficking.

### Identification of a GEF homologue

In addition to multiple Rab5 isoforms, homologues of GLUP6/GEF capable of activating Rab5 forms may also be present in developing rice endosperm. A search for VPS9 domain-containing proteins in the SALADA database (Pfam) (http://salad.dna.affrc.go.jp/CGViewer/en/cgv_search_pf.html) revealed two rice GEF genes, Os03g0262900 and Os03g0842700. The former corresponded to GLUP6/GEF while the latter encoded a novel guanine nucleotide exchange factor for Rab5, which was named Rab5-GEF2 (Supplementary Fig. S5A). Os03g0842700 (or LOC_Os03g62580.1 using the MSU rice genome annotation database) is a functional gene, as a search of the rice annotation project database (RAP-DB: http://rapdb.dna.affrc.go.jp/) revealed a corresponding cDNA (AK070821). Moreover, inspection of the RiceXPro microarray RNA expression database revealed that AK070821 were expressed in developing seeds (Supplementary Fig. S5B). The expression of this gene in the embryo after 7 DAF (days after flowering) was higher than in the endosperm.

A comparison of the amino acid sequences of AK070821 (obtained from KOME: http://cdna01.dna.affrc.go.jp/cDNA/), and the predicted primary amino acid sequence of LOC_Os03g62580.1 revealed that AK070821 was not full length and was missing about 80 amino acids at the N-terminus (Supplementary Fig. S6). To generate a functional cDNA corresponding to the LOC_Os03g62580.1 gene, the full-length open reading frame was amplified with gene specific primers (Supplementary Table 1) and single strand cDNA was reverse-transcribed from mRNA extracted from immature seeds of the wild-type plants as a template. Sequence analysis of the single amplified cDNA demonstrated that the Rab5-GEF2 cDNA possessed an ORF of 927bp which encoded 308 deduced amino acids (Supplementary Fig. S7), which coincided to the deduced amino acid sequence of the 5 exons of LOC_Os03g62580.1. Similar to GLUP6/GEF, the Rab5-GEF2 contained a conserved helical bundle domain (residues 1–101) and a conserved VPS9 domain (residues 102–245).

### Rab5-GEF2 activates Rab5s

**Fig. 6.**
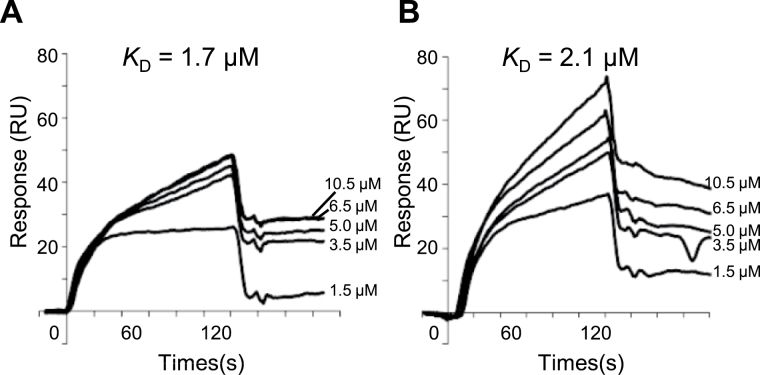
Physical interactions of GLUP4/Rab5a with Rab5-GEF2 and GLUP6/GEF. Purified GLUP4/Rab5a was immobilized on Sensor Chip CM5, and various concentrations (1.5, 3.5, 5, 6.5, and 10.5 μM) of Rab5-GEF2 (A) and GLUP6/GEF (B) were loaded onto the chip for 120 s. Each sensorgram showed the actual binding responses obtained by subtraction of the background responses. Surface plasmon resonance analysis result showed that Rab5-GEF2 and GLUP6/GEF exhibited the same patterns of interaction with GLUP4/Rab5a.

In order to determine whether Rab5-GEF2 has the potential to activate GLUP4/Rab5a, we tested whether the two proteins physically interacted using surface plasmon resonance (SPR). The *K*_D_ value of Rab5-GEF2 for GLUP4/Rab5a was measured at 1.7 μM, which is close to the *K*_D_ (2.1 μM) observed for GLUP6/GEF. Moreover the overall response over time curves observed for the Rab5-GEF2 experiment was similar to that seen when GLUP6/GEF was used as the Rab5 activator (Fig. 6). This result indicates that Rab5-GEF2 physically interacts with GLUP4/Rab5a just as efficiently as with GLUP6/GEF.

**Fig. 7.**
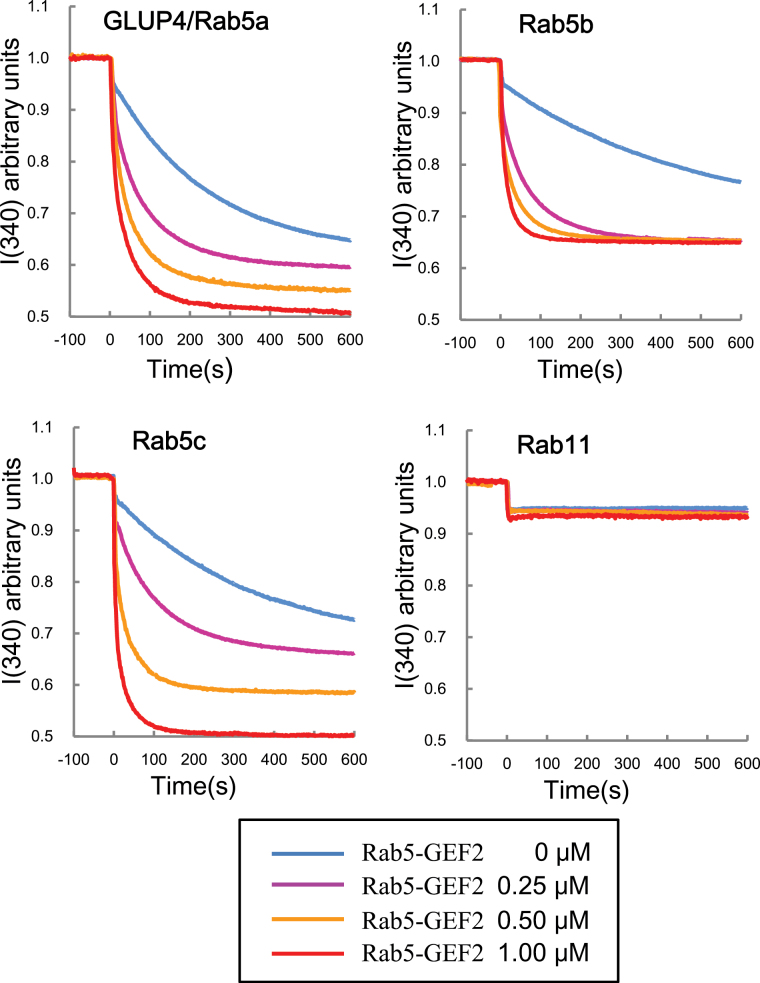
*in vitro* GEF assay of Rab5-GEF2. The conformational changes of Rab5s and Rab11 (as 1 μM GST fusion proteins) upon GDP/GMP-PNP exchange were measured by monitoring Trp autofluorescence when incubated with 0 μM (blue), 0.25 μM (magenta), 0.5μM (yellow), and 1 μM (red) GST-Rab5-GEF2. Note that GST-Rab5-GEF2 activates GLUP4/Rab5a, Rab5b, and Rab5c, but not Rab11.

In order to elucidate whether the Rab5-GEF2 can activate Rab5s including GLUP4/Rab5a, *in vitro* guanine nucleotide exchange assays were conducted. As expected, Rab5-GEF2 activated GLUP4/Rab5a, the plant-specific Rab5b, and Rab5c in a dose-dependent manner, while the control Rab11 was not activated by Rab5-GEF2 (Fig. 7). This result indicates that Rab5-GEF2 serves as a general activator of Rab5 isoforms. These results provide ample evidence that both GLUP6/GEF and Rab5-GEF2 activate multiple Rab5 proteins present in developing rice endosperm.

### The role of domains in GEFs for activity

**Fig. 8.**
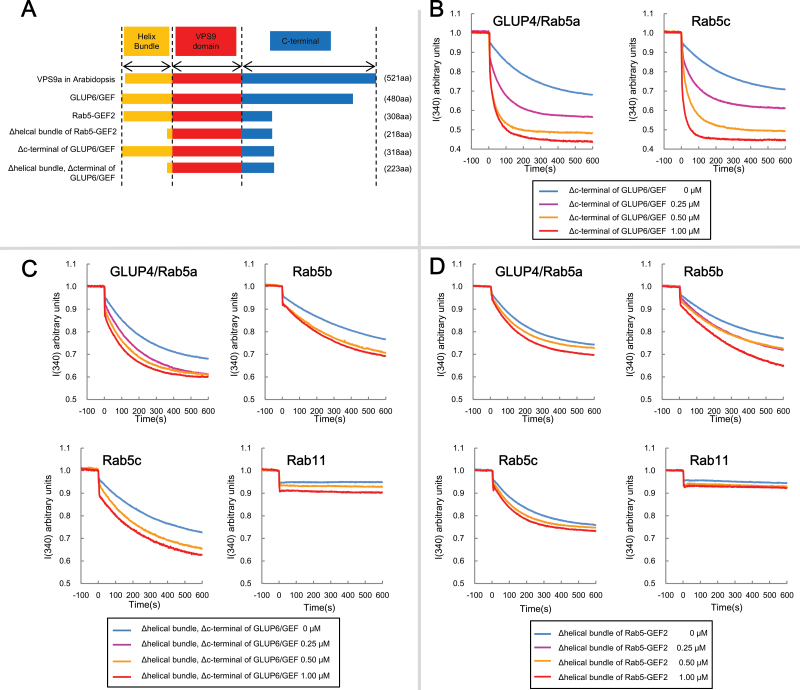
*in vitro* GEF assay of truncated GEFs. The conformational changes of Rab5s and Rab11 (as 1 μM GST fusion proteins) upon GDP/GMP-PNP exchange were measured by monitoring Trp autofluorescence when incubated with 0 μM (blue), 0.25 μM (magenta), 0.5μM (yellow), and 1 μM (red) GST-truncate GEFs. (A) Schematic structure of GEF proteins. VPS9a: Rab5 specific GEF in *Arabidopsis* (Goh *et al.*, 2007). Δhelical bundle of Rab5-GEF2: deletion of helical bundle domain sequence, 90 amino acids from Rab5-GEF2. Δc-terminal of GLUP6/GEF: deletion of C-terminal sequence, 162 amino acids from GLUP6/GEF. (B) *in vitro* GEF assay of Δc-terminal of GLUP6/GEF. Note that GST-Δc-terminal of GLUP6/GEF activates GLUP4/Rab5a and Rab5c. (C) *in vitro* GEF assay of Δhelical bundle, Δc-terminal of GLUP6/GEF. Note that GST-Δhelical bundle, Δc-terminal of GLUP6/GEF cannot activate GLUP4/Rab5a, Rab5b and Rab5c. (D) *in vitro* GEF assay of Δhelical bundle of Rab5-GEF2. Note that GST-Δhelical bundle of Rab5-GEF2 cannot activate GLUP4/Rab5a, Rab5b, and Rab5c.

The deduced primary sequence of Rab5-GEF2, 308 amino acids in length, is 172 residues shorter than GLUP6/GEF as it lacks the longer C-terminal region of GLUP6/GEF (Fig. 8A). As Rab5-GEF2 activates Rab proteins, the C-terminal region in GLUP6/GEF is likely not required for activation of the Rab5 proteins. To obtain evidence to support this view, a truncated form of GLUP6/GEF (ΔC-GLUP6/GEF) was constructed, which lacks 162 amino acids at the C-terminal region (Fig. 8A). As shown in Fig. 8B, ΔC-GLUP6/GEF was capable of activating GLUP4/Rab5a and Rab5c in a dose-dependent manner, indicating that the C-terminal region is not required for activation of Rab5 proteins.

The mammalian Rab5 GEF harbours a helical bundle (HB) domain consisting of conserved ∼100 amino acids at the N-terminal end of the Vps9 domain, which stabilizes the Vps9 domain (Delprato *et al.*, 2004). In order to evaluate the influence of the HB domain on GEF activity for rice Rab5 GEFs, truncated ΔHB forms for GLUP6/GEF and Rab5-GEF2 were constructed, lacking 90 amino acids of the N-termini (Fig. 8A). When tested in the *in vitro* GEF assay, the activity of ΔHB/Rab5-GEF2 and ΔHB/ΔC-GLUP6/GEF to all tested Rab5 proteins was greatly reduced (Fig. 8C, D). Liu *et al.* (2013) similarly reported that a truncated Rab5-GEF2 protein lacking the N-terminal 90 amino acids exhibited weak physical interaction with Rab5 proteins. Overall, the HB domain in GEF proteins is indispensable for the activation of the Rab5 family.

## Discussion

### Regulation for biosynthesis, intracellular transport, and accumulation of glutelin

The study of the *glup* mutant lines, which accumulate abnormally high amounts of proglutelin, have identified the function of several factors that participate in the biosynthesis, intracellular transport, maturation and packaging of glutelins into PSVs. Further gene-gene interaction analysis between proglutelin accumulating mutations (Ueda *et al.*, 2010) agrees well with the results obtained by biochemical and histochemical analyses of the mutations. Within the ER, protein disulfide isomerase-like (PDIL) 1-1 is essential for the segregation of proglutelin and prolamine polypeptides within the ER lumen and the maturation of proglutelin, especially when its rate of synthesis significantly exceeds its export from the ER (Takemoto *et al.*, 2002; Satoh-Cruz *et al.*, 2010a). GLUP4/Rab5a and its cognate GDP/GTP nucleotide exchange factor, GLUP6/GEF, participate in the intracellular transport of the proglutelin from the Golgi apparatus to the PSV (Fukuda *et al.*, 2011, 2013). The vacuolar processing enzyme, which cleaves proglutelin to two subunits within the PSV, plays an important role in the formation of the glutelin-containing crystalline structures observed in PSV micrographs (Kumamaru *et al.*, 2010). Additionally, Sar1, a small GTPase, acting as a molecular switch to regulate the assembly of coat protein complex II (COPII), plays a role in the transport of proglutelin and α-globulin from the ER to the Golgi apparatus (Tian *et al.*, 2013).

Loss of GLUP4/Rab5a or GLUP6/GEF activities result in the abnormal accumulation of proglutelin and the appearance of PMBs (Fukuda *et al.*, 2011, 2013). As demonstrated earlier by Liu *et al.* (2013), the combined loss of both Rab5 and GEF magnified these phenotypic changes. We also demonstrated in the present study that rice contains multiple Rab5 and GEF isoforms that are capable of biochemically interacting with each other (Figs 5, 7). These results provide the causal basis for the apparent synergistic interaction between the *glup4/rab5a* and *glup6/gef* mutations resulting in a larger proportion of proglutelin accumulation and more prominent PMBs than that seen in either parent alone. Overall, the available evidence suggests that multiple Rab5 and GEF isoforms participate in the intracellular transport of the proglutelin from the Golgi apparatus to the PSV.

Mutations in the *GPA3* gene also result in developing rice seeds having the same phenotypes (i.e. proglutelin accumulation and PMB formation) as *glup4* and *glup6* (Ren *et al.*, 2014). *GPA3* gene codes for a kelch-repeat protein, which interacts with OsVPS9A (GLUP6/GEF) and participates in anterograde post-Golgi trafficking of proglutelin. Double recessive types of kelch-repeat protein and OsRAB5A or OsVPS9A exhibit the same synergistic genetic interactions as Rab5 and GEF indicating that they are involved in the same trafficking events (Ren *et al.*, 2014). In addition to these mutant lines, a dominant *Glup5* was identified and genetically classified to the same class as *glup4* and *glup6* (Ueda *et al.*, 2010). Although the *GLUP5* gene has yet to be identified, the available evidence indicates that additional unknown factors participate in the intracellular transport of the proglutelin from the Golgi apparatus.

### Rice developed a different Rab5-GEF system from *Arabidopsis*

Several GEFs for Rab5 had been documented including the yeast Vps9p, the mammalian Rabex-5, RIN-1, RIN-2, RIN-3, RME-6, Alsin, and ALS2CL (Carney *et al.*, 2006) and the *Arabidopsis* VPS9a and VPS9b isoforms (Goh *et al.*, 2007; Vernoud *et al.*, 2003). The *Arabidopsis* VPS9a is capable of activating the three Rab5 family proteins and is the major GEF activity, whereas *VPS9b* is expressed at very low levels in vegetative tissues when viewed by RT-PCR analysis or by analysis of the public expression database. This dominant role of VPS9a is also supported by the severe growth phenotypes exhibited by T-DNA insertion mutants of VPS9a. These lines of evidence point to VPS9a as representing the major GEF activity for Rab5 in *Arabidopsis* (Goh *et al.*, 2007). Based on neighbor-joining tree analysis, GLUP6/GEF is the rice orthologue of VPS9a. The corresponding counterparts of Rab5-GEF2 and VPS9b, however, do not exist in *Arabidopsis* and rice, respectively (Supplementary Fig. S5A).

Mammalian cells utilize several GEF isoforms to activate Rab5 at different steps of the endocytic pathway. RME6 participates in formation of clathrin-coated vesicles for endocytosis at the plasma membrane (Sato *et al.*, 2005). Rabex-5 complexed with the adaptor protein Rabaptin-5 is essential for homotypic fusion between early endosomes (Horiuchi *et al.*, 1997; McBride *et al.*, 1999; Lippe *et al.*, 2001) while RIN1 stimulates Rab5-dependent endosome fusion and epidermal growth factor receptor-mediated endocytosis (Tall *et al.*, 2001). Compared with animals and *Arabidopsis*, rice appears to have developed a more generalized Rab5-GEF system in having redundant GEFs and Rab5 activities participate in the transport of proglutelins from the Golgi apparatus to the PSV.

### Helical bundle domain of GEF is essential for the physical interaction with Rab5 family

In addition to the conserved VPS9 domain, many Rab5 GEFs share a second conserved helical bundle (HB) domain (Burd *et al.*, 1996; Horiuchi *et al.*, 1997). This HB domain, originally identified in Rabex-5 3-D structure, stabilizes the Vps9 domain (Delprato *et al.*, 2004). Deletion of the HB domain in Rab5-GEF2 and GLUP6/GEF resulted in loss of ability to activate Rab5 members (Fig. 8C, D), although the truncated Rab5-GEF2 lacking the HB domain weakly interacted with GLUP4/Rab5a (Liu *et al.*, 2013). The truncated GLUP6/GEF containing the deleted HB domain and C-terminal region was also unable to activate Rab5s, although deletion of the C-terminal region in GLUP6/GEF had no significant effect on the activation of Rab5s (Fig. 8). The HB domain is also conserved in VPS9a and Rabex-5 (Uejima *et al.*, 2010) with the sequence similarity in the HB domain of Rab5-GEF2 to GLUP6/GEF, VPS9a, VPS9b, and Rabex-5 being 59.1%, 65.3%, 64.4%, and 40.9%, respectively (Supplementary Fig. S8). These facts suggest that the HB domain is essential for the physical interaction of GEFs with Rab5 proteins.

## Supplementary data

Supplementary data are available at *JXB* online.

Supplementary Fig. S1. Immunofluorescence microscopy of the endosperm in the double recessive type of *glup4/rab5a* and *glup6/gef*.

Supplementary Fig. S2. Transmission electron microscopy of endosperm in the *glup4/rab5a* and *glup6/gef*.

Supplementary Fig. S3. Immuno-electron microscopy of endosperm from the double recessive type of *glup4/rab5a* and *glup6/gef*.

Supplementary Fig. S4. Expression analysis of Rab5 homologues in rice.

Supplementary Fig. S5. Analysis of GEF genes for Rab5 in rice.

Supplementary Fig. S6. Alignment of amino acid sequence of transcripts from Os03g0842700.

Supplementary Fig. S7. cDNA and the deduced amino acid sequences corresponding to Os03g0842700.

Supplementary Fig. S8. Alignment of helical bundle domain from different GEFs.

Supplementary Table 1. List of PCR primers.

Supplementary Data

## Abbreviations:

COP: coat protein
CTAB: cetyltrimethylammonium bromide
DDBJ: DNA data bank of Japan
esp: endosperm storage protein
ER: endoplasmic reticulum
GEF: guanine nucleotide exchange factor
glup: glutelin precursor
GMP-PNP: 5′-guanylyl imidodiphosphate
HB: helical bundle
KOME: knowledge-based *Oryza* molecular biological encyclopedia
MNU: *N*-methyl-*N*-nitrosourea
PB: protein body
PDIL: protein disulfide isomerase-like
PMBs: paramural bodies
PSV: protein storage vacuole
RAP-DB: rice annotation project database
PVC: prevacuolar compartment
RiceXRro: rice expression profile database
RT: reverse transcript
SALADA: alignment diagram and the association dendrogram
SPR: surface plasmon resonance
WAF: week after flowering.

## References

[CIT0001] Bolte S Brown S Satiat-Jeunemaitre B 2004 The N-myristoylated Rab-GTPase m-Rabmc is involved in post-Golgi trafficking events to the lytic vacuole in plant cells. Journal of Cell Science 117, 943−954. 14762108 10.1242/jcs.00920

[CIT0002] Burd CG Mustol PA Schu PV Emr SD 1996 A yeast protein related to a mammalian Ras-binding protein, Vps9p, is required for localization of vacuolar proteins. Molecular and Cellular Biology 16, 2369−2377. 8628304 10.1128/mcb.16.5.2369 PMC231225

[CIT0003] Carney DS Davies BA Horazdovsky BF 2006 Vps9 domain-containing proteins: activators of Rab5 GTPases from yeast to neurons. Trends in Cell Biology 16, 27−35. 16330212 10.1016/j.tcb.2005.11.001

[CIT0004] Delprato A Merithew E Lambright DG 2004 Structure, exchange determinants, and family-wide rab specificity of the tandem helical bundle and Vps9 domains of Rabex-5. Cell 118, 607−617. 15339665 10.1016/j.cell.2004.08.009

[CIT0005] Ebine K Fujimoto M Okatani Y 2011 A membrane trafficking pathway regulated by the plant-specific RAB GTPase ARA6. Nature Cell Biology 13, 853−859. 21666683 10.1038/ncb2270

[CIT0006] Fukuda M Satoh-Cruz M Wen L 2011 The small GTPase Rab5a is essential for intracellular transport of proglutelin from the Golgi apparatus to the protein storage vacuole and endosomal membrane organization in developing rice endosperm. Plant Physiology 157, 632−644. 21825104 10.1104/pp.111.180505 PMC3192576

[CIT0007] Fukuda M Wen L Satoh-Cruz M 2013 A guanine nucleotide exchange factor for Rab5 proteins is essential for intracellular transport of the proglutelin from the Golgi apparatus to the protein storage vacuole in rice endosperm. Plant Physiology 162, 663−674. 23580596 10.1104/pp.113.217869 PMC3668061

[CIT0008] Goh T Uchida W Arakawa S 2007 VPS9a, the common activator for two distinct types of Rab5 GTPases, is essential for the development of *Arabidopsis thaliana* . The Plant Cell 19, 3504−3515. 18055610 10.1105/tpc.107.053876 PMC2174884

[CIT0009] Haas TJ Sliwinski MK Martinez DE Preuss M Ebine K Ueda T Nielsen E Odorizzi G Otegui MS 2007 The *Arabidopsis* AAA ATPase SKD1 is involved in multivesicular endosome function and interacts with its positive regulator LYST-INTERACTING PROTEIN5. The Plant Cell 19, 1295−1312. 17468262 10.1105/tpc.106.049346 PMC1913750

[CIT0010] Horiuchi H Lippe R McBride HM 1997 A novel Rab5 GDP/GTP exchange factor complexed to Rabaptin-5 links nucleotide exchange to effector recruitment and function. Cell 90, 1149−1159. 9323142 10.1016/s0092-8674(00)80380-3

[CIT0011] Kotzer AM Brandizzi F Neumann U Paris N Moore I Hawes C 2004 AtRabF2b (Ara7) acts on the vacuolar trafficking pathway in tobacco leaf epidermal cells. Journal of Cell Science 117, 6377−6389. 15561767 10.1242/jcs.01564

[CIT0012] Krishnan HB Franceschi VR Okita TW 1986 Immunochemical studies on the role of the Golgi complex in protein-body formation in rice seeds. Planta 169, 471−480. 24232753 10.1007/BF00392095

[CIT0013] Kumamaru T Ogawa M Satoh H Okita TW 2007 Protein body biogenesis in cereal endosperms. In: Olsen OA, ed. Endosperm—Development and Molecular Biology , Vol. 8 Berlin: Springer-Verlag, 141−158.

[CIT0014] Kumamaru T Uemura Y Inoue Y Takemoto Y Siddiqui SU Ogawa M Hara-Nishimura I Satoh H 2010 Vacuolar processing enzyme plays an essential role in the crystalline structure of glutelin in rice seed. Plant and Cell Physiology 51, 38−46. 19933265 10.1093/pcp/pcp165

[CIT0015] Lee GJ Sohn EJ Lee MH Hwang I 2004 The *Arabidopsis* rab5 homologs rha1 and ara7 localize to the prevacuolar compartment. Plant and Cell Physiology 45, 1211−1220. 15509844 10.1093/pcp/pch142

[CIT0016] Lippe R Miaczynska M Rybin V Runge A Zerial M 2001 Functional synergy between Rab5 effector Rabaptin-5 and exchange factor Rabex-5 when physically associated in a complex. Molecular Biology of the Cell 12, 2219−2228. 11452015 10.1091/mbc.12.7.2219 PMC55678

[CIT0017] Liu F Ren Y Wang Y 2013 OsVPS9A functions cooperatively with OsRAB5A to regulate post-Golgi dense vesicle-mediated storage protein trafficking to the protein storage vacuole in rice endosperm cells. Molecular Plant 6, 1918−1932. 23723154 10.1093/mp/sst081

[CIT0018] McBride HM Rybin V Murphy C Giner A Teasdale R Zerial M 1999 Oligomeric complexes link Rab5 effectors with NSF and drive membrane fusion via interactions between EEA1 and syntaxin 13. Cell 98, 377−386. 10458612 10.1016/s0092-8674(00)81966-2

[CIT0019] Murray MG Thompson WF 1980 Rapid isolation of high molecular weight plant DNA. Nucleic Acids Research 8, 4321−4325. 7433111 10.1093/nar/8.19.4321 PMC324241

[CIT0020] Nagamine A Matsusaka H Ushijima T Kawagoe Y Ogawa M Okita TW Kumamaru T 2011 A role for the cysteine-rich 10kDa prolamin in protein body I formation in rice. Plant and Cell Physiology 52, 1003−1016. 21521743 10.1093/pcp/pcr053 PMC3110882

[CIT0021] Ren Y Wang Y Liu F 2014 Glutelin precursor accumulation3 encodes a regulator of post-Golgi vesicular traffic essential for vacuolar protein sorting in rice endosperm. The Plant Cell 26, 410−425. 24488962 10.1105/tpc.113.121376 PMC3963586

[CIT0022] Sato M Sato K Fonarev P Huang CJ Liou W Grant BD 2005 *Caenorhabditis elegans* RME-6 is a novel regulator of RAB-5 at the clathrin-coated pit. Nature Cell Biology 7, 559−569. 15895077 10.1038/ncb1261 PMC1398054

[CIT0023] Satoh-Cruz M Crofts AJ Takemoto-Kuno Y Sugino A Washida H Crofts N Okita TW Ogawa M Satoh H Kumamaru T 2010 *a* . Protein disulfide isomerase like 1-1 participates in the maturation of proglutelin within the endoplasmic reticulum in rice endosperm. Plant and Cell Physiology 51, 1581−1593. 20627947 10.1093/pcp/pcq098

[CIT0024] Satoh-Cruz M Fukuda M Ogawa M Satoh H Kumamaru T 2010 *b* . *Glup4* gene encodes small GTPase, Rab5a in rice. Rice Genetics Newsletter 25, 48−49.

[CIT0025] Sohn EJ Kim ES Zhao M 2003 Rha1, an *Arabidopsis* Rab5 homolog, plays a critical role in the vacuolar trafficking of soluble cargo proteins. The Plant Cell 15, 1057−1070. 12724533 10.1105/tpc.009779 PMC153716

[CIT0026] Stierhof YD El Kasmi F 2010 Strategies to improve the antigenicity, ultrastructure preservation and visibility of trafficking compartments in *Arabidopsis* tissue. European Journal of Cell Biology 89, 285−297. 20106548 10.1016/j.ejcb.2009.12.003

[CIT0027] Takemoto Y Coughlan SJ Okita TW Satoh H Ogawa M Kumamaru T 2002 The rice mutant *esp2* greatly accumulates the glutelin precursor and deletes the protein disulfide isomerase. Plant Physiology 128, 1212−1222. 11950970 10.1104/pp.010624 PMC154249

[CIT0028] Tall GG Barbieri MA Stahl PD Horazdovsky BF 2001 Ras-activated endocytosis is mediated by the Rab5 guanine nucleotide exchange activity of RIN1. Developmental Cell 1, 73−82. 11703925 10.1016/s1534-5807(01)00008-9

[CIT0030] Tian L Dai LL Yin ZJ Fukuda M Kumamaru T Dong XB Xu XP Qu le Q 2013 Small GTPase Sar1 is crucial for proglutelin and alpha-globulin export from the endoplasmic reticulum in rice endosperm. Journal of Experimental Botany 64, 2831−2845. 23682119 10.1093/jxb/ert128 PMC3697955

[CIT0031] Ueda T Uemura T Sato MH Nakano A 2004 Functional differentiation of endosomes in *Arabidopsis* cells. The Plant Journal 40, 783−789. 15546360 10.1111/j.1365-313X.2004.02249.x

[CIT0032] Ueda T Yamaguchi M Uchimiya H Nakano A 2001 Ara6, a plant-unique novel type Rab GTPase, functions in the endocytic pathway of *Arabidopsis thaliana* . The EMBO Journal 20, 4730−4741. 11532937 10.1093/emboj/20.17.4730 PMC125591

[CIT0033] Ueda Y Satoh-Cruz M Matsusaka H Takemoto-Kuno Y Fukuda M Okita TW Ogawa M Satoh H Kumamaru T 2010 Gene-gene interactions between mutants that accumulate abnormally high amounts of proglutelin in rice seed. Breeding Science 60, 568−574.

[CIT0034] Uejima T Ihara K Goh T Ito E Sunada M Ueda T Nakano A Wakatsuki S 2010 GDP-bound and nucleotide-free intermediates of the guanine nucleotide exchange in the Rab5.Vps9 system. Journal of Biological Chemistry 285, 36689−36697. 20833725 10.1074/jbc.M110.152132 PMC2978598

[CIT0035] Ushijima T Matsusaka H Jikuya H Ogawa M Satoh H Kumamaru T 2011 Genetic analysis of cysteine-poor prolamin polypeptides reduced in the endosperm of the rice *esp1* mutant. Plant Science 181, 125−131. 21683877 10.1016/j.plantsci.2011.04.011

[CIT0036] Vernoud V Horton AC Yang Z Nielsen E 2003 Analysis of the small GTPase gene superfamily of Arabidopsis. Plant Physiology 131, 1191−1208. 12644670 10.1104/pp.013052 PMC166880

[CIT0037] Wang Y Ren Y Liu X 2010 OsRab5a regulates endomembrane organization and storage protein trafficking in rice endosperm cells. The Plant Journal 64, 812−824. 21105928 10.1111/j.1365-313X.2010.04370.x

[CIT0038] Woollard AA Moore I 2008 The functions of Rab GTPases in plant membrane traffic. Current Opinion in Plant Biology 11, 610−619. 18952493 10.1016/j.pbi.2008.09.010

[CIT0039] Yamagata H Sugimoto T Tanaka K Kasai Z 1982 Biosynthesis of storage proteins in developing rice seeds. Plant Physiology 70, 1094−1100. 16662620 10.1104/pp.70.4.1094 PMC1065832

[CIT0040] Yamagata H Tanaka K 1986 The site of synthesis and accumulation of storage proteins. Plant and Cell Physiology 27, 135−145.

