# Supplementary material for: Guanine nucleotide exchange factor 2 for Rab5 proteins coordinated with GLUP6/GEF regulates the intracellular transport of the proglutelin from the Golgi apparatus to the protein storage vacuole in rice endosperm
Source: J Exp Bot. 2015 Jul 1;66(20):6137–47. doi: 10.1093/jxb/erv325 (PMC4588877; doi:10.1093/jxb/erv325)
Supplement: Supplementary Data [file supp_66_20_6137__index.html]

Guanine nucleotide exchange factor 2 for Rab5 proteins coordinated with GLUP6/GEF regulates the intracellular transport of the proglutelin from the Golgi apparatus to the protein storage vacuole in rice endosperm — Guanine nucleotide exchange factor 2 for Rab5 proteins coordinated with GLUP6/GEF regulates the intracellular transport of the proglutelin from the Golgi apparatus to the protein storage vacuole in rice endosperm — Supplementary Data 

# Guanine nucleotide exchange factor 2 for Rab5 proteins coordinated with GLUP6/GEF regulates the intracellular transport of the proglutelin from the Golgi apparatus to the protein storage vacuole in rice endosperm

## Supplementary Data

Data files

- Supplementary Data - Supplementary Data
