# Supplementary material for: Guanine nucleotide exchange factor 2 for Rab5 proteins coordinated with GLUP6/GEF regulates the intracellular transport of the proglutelin from the Golgi apparatus to the protein storage vacuole in rice endosperm
Source: J Exp Bot. 2015 Jul 1;66(20):6137–47. doi: 10.1093/jxb/erv325 (PMC4588877; doi:10.1093/jxb/erv325)
Supplement: Supplementary Data [file supp_erv325_jexbot145672_file001.pdf]

Guanine nucleotide exchange factor 2 for Rab5 proteins coordinated with GLUP6/GEF regulates the intracellular transport of the proglutelin from the Golgi apparatus to the protein storage vacuole in rice endosperm

Liuying Wen, Masako Fukuda, Mariko Sunada, Sonoko Ishino, Yoshizumi Ishino, Thomas W. Okita, Masahiro Ogawa, Takashi Ueda, and Toshihiro Kumamaru

Supplemental Files

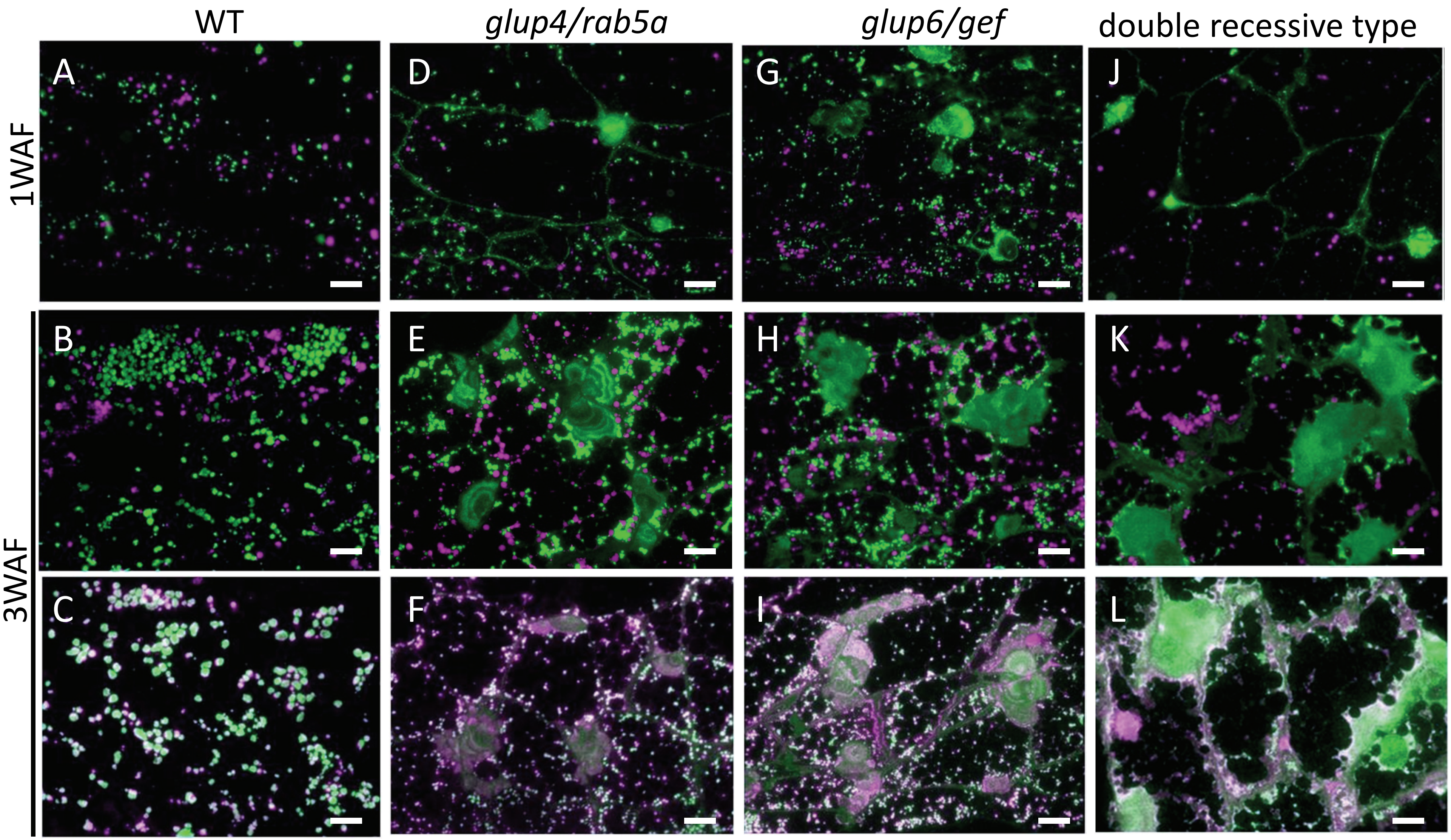

Supplemental Figure S1. Immunofluorescence microscopy of the endosperm in the double recessive type of *glup4/rab5a* and *glup6/gef*.
A to C: wild type, D to F: *glup4/Rab5a*, G to I: *glup6/gef*, J to L: the double recessive type from the crossing of EM425 and EM939. A, D, G, J and B, C, E, F, H, I, K, L depict sections of developing at 1 WAF and 3 WAF, respectively. A, B, D, E, G, H, J, and K: Secondary antibodies labeled with rhodamine (magenta) and fluorescein isothiocyanate (FITC: green) were used to visualize the reaction of prolamine and glutelin antibodies, respectively. C, F, I, and L: Secondary antibodies labeled with rhodamine (magenta) and fluorescein isothiocyanate (FITC: green) were used to visualize the reaction of α-globulin and glutelin antibodies, respectively. Bars = 10 μm.

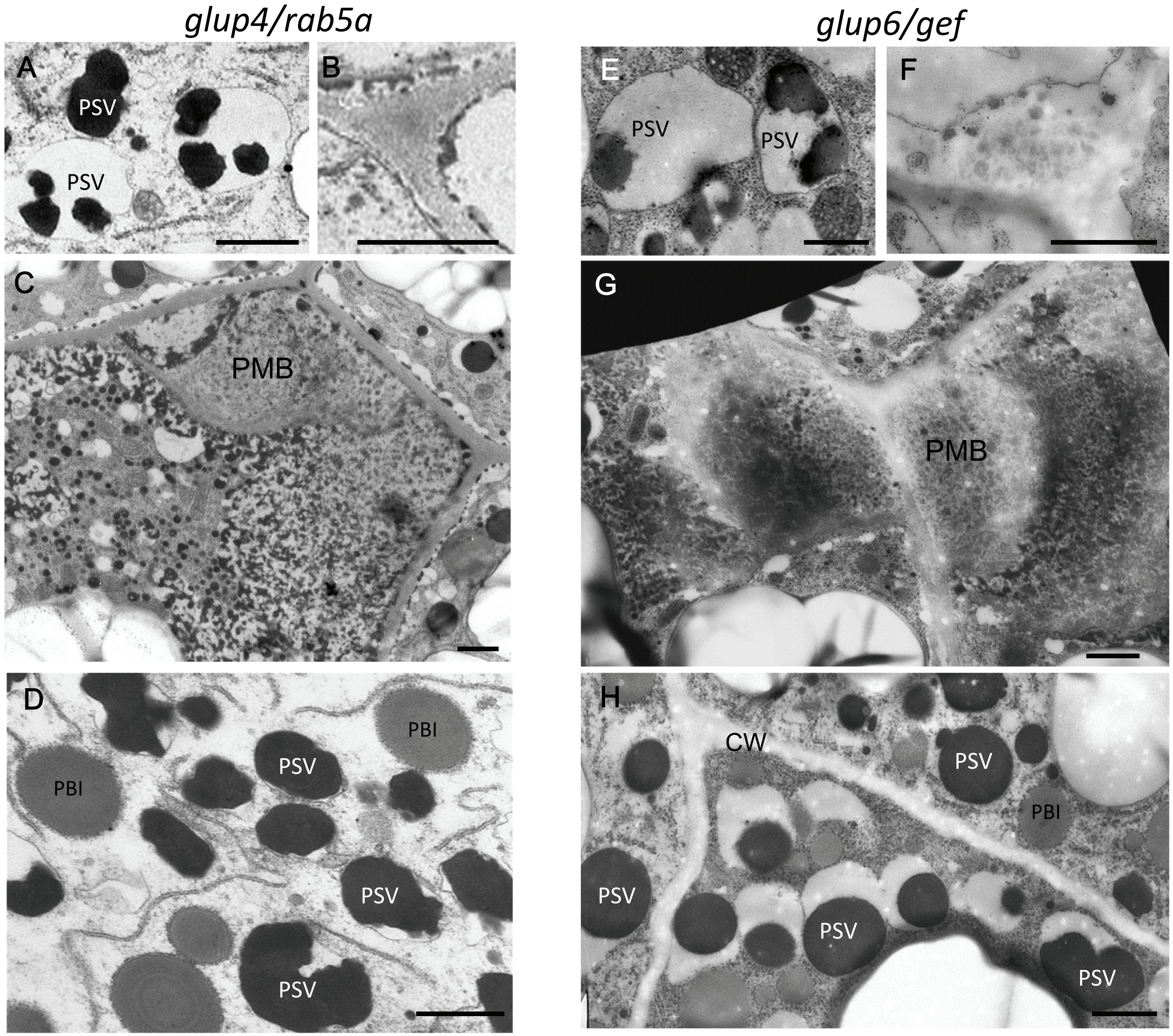

Supplemental Figure S2. Transmission electron microscopy of endosperm in the *glup4/rab5a* and *glup6/gef*.
A to D: *glup4/rab5a*, EM956, E to H: *glup6/gef*, EM939. A, B, E, and F, C and G, D and H depict sections of developing endosperm at 1 WAF, 2 WAF, and 3 WAF, respectively. Bars = 1 µm.

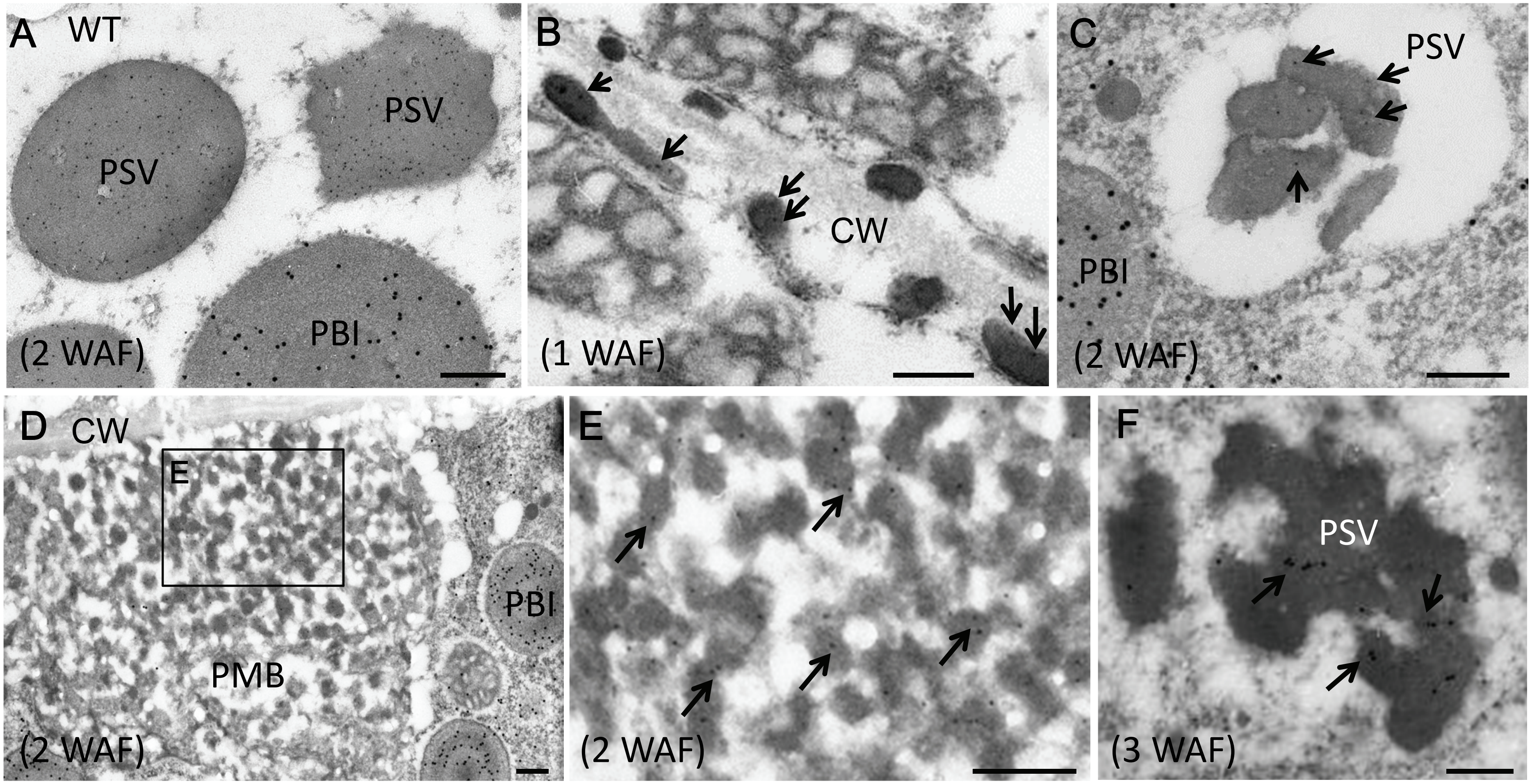

Supplemental Figure S3. Immuno-electron microscopy of endosperm from the double recessive type of *glup4/rab5a* and *glup6/gef*.

A: wild type, B to F: double recessive type from the crossing of EM425 (*glup4/rab5a*) and EM939 (*glup6/gef*). B: 1 WAF; A, C, D, and E: 2 WAF; F: 3 WAF. E and F, are enlarged images of the square areas in D and Fig. 3J. A to E: Gold particles of 5 nm and 15 nm indicate the reaction of glutelin and prolamine antibodies, respectively. F: Gold particles of 15 nm and 5 nm indicate the reaction of glutelin and prolamine antibodies, respectively. Arrows in B, C, E, and F show the reaction of glutelin antibodies. Bars = 200 nm in A to F.

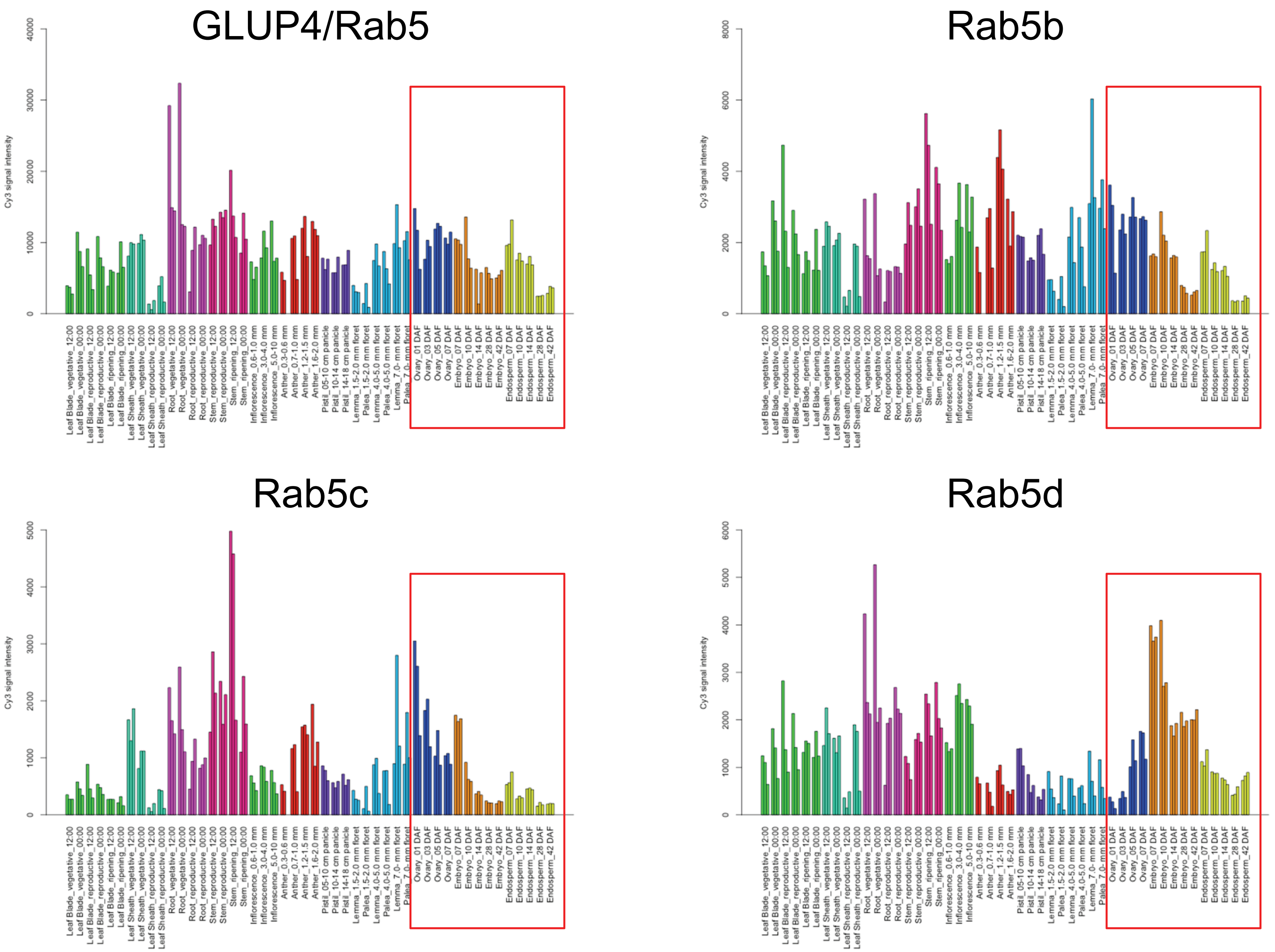

Supplemental Figure S4. Expression analysis of Rab5 homologues in rice.
Data was collected from the rice global gene expression profile database, RiceXPro (http://ricexpro.dna.affrc.go.jp/pro/). Red box indicates the expression level in seeds.

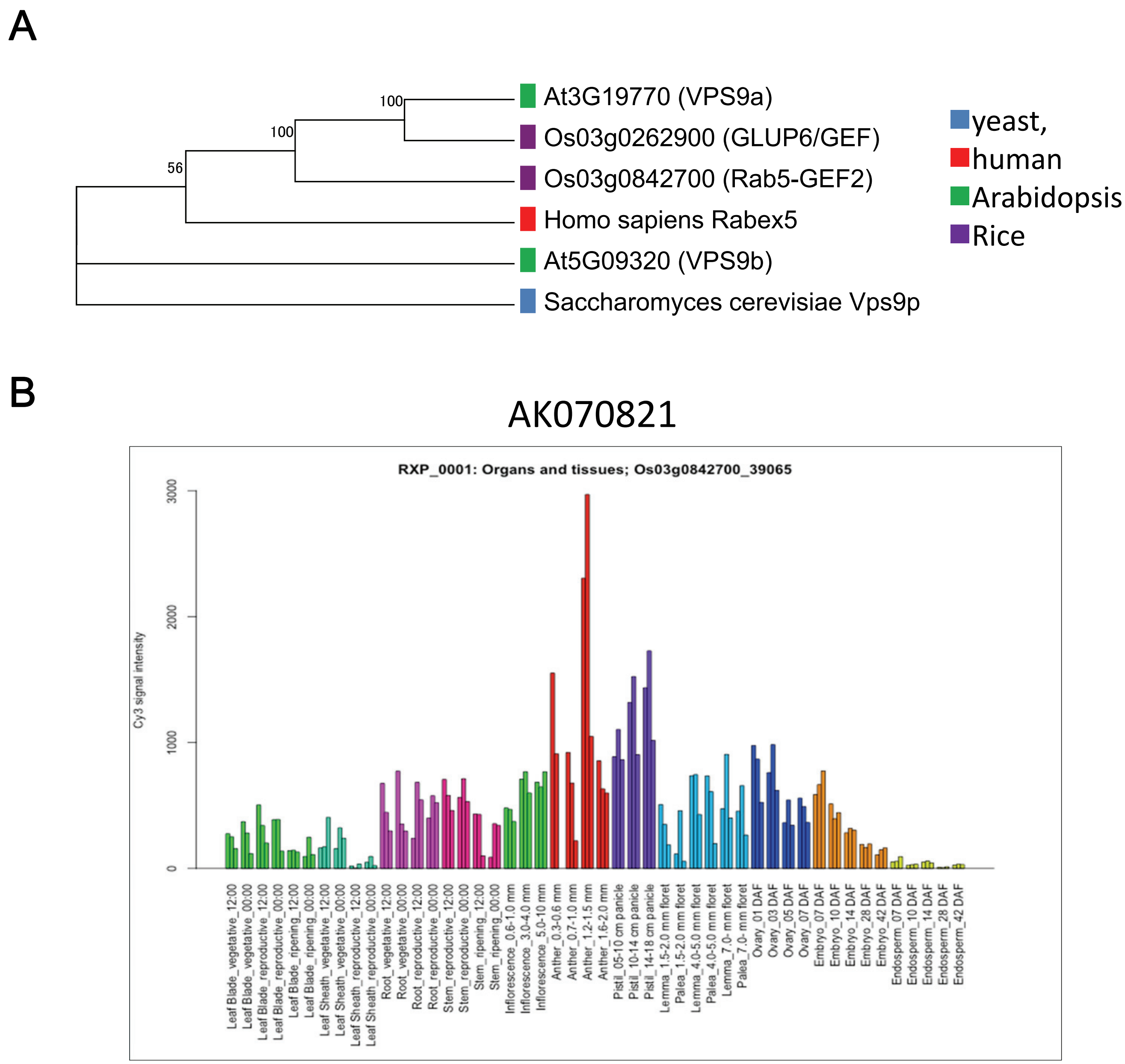

Supplemental Figure S5. Analysis of GEF genes for Rab5 in rice.
A: Neighbor-joining tree of GEF proteins from different species.
B: *Rab5-GEF2* gene transcripts. Data was collected from the rice global gene expression profile database, RiceXPro.

```
CLUSTAL 2.1 Multiple Sequence Alignments

Sequence 1: GLUP6/GEF          480 aa
Sequence 2: AK070821           218 aa
Sequence 3: LOC_Os03g62580.1   308 aa


AK070821                ------------------------------------------------------------
LOC_Os03g62580.1        ------MESPTSPASRLDFYDFIGRMRRPAAADLFHSIRSFLASLSQGGEPNAEVDGGRV 54
GLUP6/GEF               MDGGGGGDAFGSATAPLAWHDFLERMRQPSAADFVKSIKGFIVTFSN-RAPDPEHDSAAV 59

AK070821                ------------------------------------MTKLFDRAFASSAEDVKSDMEISE 24
LOC_Os03g62580.1        QTFFAEMETAIRDHPLWANATNQEIDNALEGLEKYIMTKLFDRAFASSAEDVKSDMEISE 114
GLUP6/GEF               QEFLENMEGAFRAHTPWAGSSEEELESAGEGLEKYVMTKLFNRVFASVPEDVKSDEELFE 119
                                                            *****:*.*** .****** *: *

AK070821                KIGLLQHFVRPHHLDIPKLLHNEAAWLLAVKELQKINSFKSPREKLSCIMSCCQVINNLL 84
LOC_Os03g62580.1        KIGLLQHFVRPHHLDIPKLLHNEAAWLLAVKELQKINSFKSPREKLSCIMSCCQVINNLL 174
GLUP6/GEF               KMSLLQQFIRPENLDIKPEYQSETSWLLAQKELQKINMYKAPRDKLACILNCCKVINNLL 179
                        *:.***:*:**.:***    :.*::**** ******* :*:**:**:**:.**:******

AK070821                LNVSMSNDRTLSGADDFLPILIYITIKANPPQLHSNLKFIQLFRRETRLISEVEYYLTNL 144
LOC_Os03g62580.1        LNVSMSNDRTLSGADDFLPILIYITIKANPPQLHSNLKFIQLFRRETRLISEVEYYLTNL 234
GLUP6/GEF               LNASIVSNENPPGADEFLPVLIYVTIKANPPQLHSNLLYIQRYRRQSRLVSEAQYFFTNI 239
                        **.*: .:.. .***:***:***:************* :** :**::**:**.:*::**:

AK070821                ISAKMFIMNVNGHSLSMEESVFQTHMESAR---LGNHISVASTNSSQGLGTSTP-GQNEE 200
LOC_Os03g62580.1        ISAKMFIMNVNGHSLSMEESVFQTHMESAR---LGNHISVASTNSSQGLGTSTP-GQNEE 290
GLUP6/GEF               LSAESFIWNIDGESLSMDERDFQKKMDLARERLLGLSASSENQDNQNNLDVREQKSQTLK 299
                        :**: ** *::*.****:*  **.:*: **   **   *  . :..:.*..    .*. :

AK070821                SGDTEGLFLTLVDQFVSE------------------------------------------ 218
LOC_Os03g62580.1        SGDTEGLFLTLVDQFVSE------------------------------------------ 308
GLUP6/GEF               ASRDSDVNLSLKDNFQGPGLEMRRDSDASSNPVERVQSISDLEKKGAAELLKDDDLNKKI 359
                        :.  ..: *:* *:* .

AK070821                ------------------------------------------------------------
LOC_Os03g62580.1        ------------------------------------------------------------
```

```
GLUP6/GEF           QEYPFLFARSGDLTVADVENLLNSYKQLVLKYVALSQGMGINLENPPVQSMQTVSDLVES 419

AK070821            ------------------------------------------------------------
LOC_Os03g62580.1    ------------------------------------------------------------
GLUP6/GEF           EEPKNVKNAVNFSEGSSKTSDDIKNDTLYSEVDNTGTQQTAVDPSYQKAQQDEASDQPEH 479

AK070821            -
LOC_Os03g62580.1    -
GLUP6/GEF           A 480
```

Supplemental Figure. S6. Alignment of amino acid sequence of transcripts from Os03g0842700.

Amino acids highlighted with yellow and blue indicate the helical bundle domain and VPS9 domain of GLUP6/GEF. Sequence alignment of the transcripts from Os03g0842700 gene and GLUP6/GEF were performed alignment by Clustal IX 2.1.

(A) DNA sequence corresponding to Os03g0842700 [GEF2 cDNA sequence]

ATGGAGAGCCCCACGTCGCCGGCGTCGCGGCTGGACTTCTACGACTTCATCGGCC
GCATGCGCCGCCCCGCCGCCGCCGACCTCTTCCACTCCATCAGGAGCTTCCTCGC
GTCCCTCTCCCAGGGCGGCGAGCCCAACGCCGAGGTCGACGGCGGCAGGGTCCA
GACCTTCTTCGCGGAGATGGAGACCGCCATCAGGGACCACCCGCTTTGGGCCAAT
GCCACCAATCAGGAAATCGACAACGCGCTCGAGGGCTTGAGAAGTATATCATGAC
CAAATTGTTCGATCGAGCCTTCGCTTCATCCGCGGAGGATGTGAAATCCGACATGG
AGATTTCGGAGAAGATTGGTCTCTTGCAGCACTTTGTCAGGCCTCATCACTTGGACA
TACCCAAGCTTCTGCACAATGAGGCAGCGTGGCTGCTTGCAGTTAAAGAGTTGCAA
AAGATTAATTCCTTCAAATCACCACGAGAAAAGCTTAGTTGCATCATGAGCTGTTGC
CAAGTCATCAATAACTTGCTGCTAAATGTGTCAATGTCAAATGATCGAACACTATCAG
GGGCTGATGATTTTCTTCCTATTCTTATTTACATTACAATCAAGGCCAATCCTCCTCA
GTTGCACTCAAATCTAAAGTTTATTCAGCTCTTCAGAAGAGAAACAAGGCTTATCTCT
GAAGTCGAATACTATCTCACGAACCTCATTTCAGCAAAGATGTTTATAATGAATGTTA
ATGGACACTCACTGTCCATGGAGGAAAGTGTGTTCCAGACACATATGGAGTCTGCA
AGACTTGGTAACCACATATCTGTTGCTAGCACTAATAGTTCACAAGGATTGGGTACA
TCCACACCAGGACAGAACGAGGAATCTGGTGATACAGAAGGTCTGTTTTTAACATTA
GTGGATCAGTTTGTTTCAGAATAA (927bp)

1st Exon 2nd Exon 3rd Exon 4th Exon 5th Exon

(B) Deduced amino acid sequences corresponding to Os03g0842700

MESPTSPASRLDFYDFIGRMRRPAAADLFHSIRSFLASLSQGGEPNAEVDGGRVQTFFA
EMETAIRDHPLWANATNQEIDNALEGLEKYIMTKLFDRAFASSAEDVKSDMEISEKIGLL
QHFVRPHHLDIPKLLHNEAAWLLAVKELQKINSFKSPREKLSCIMSCCQVINNLLLNVSM
SNDRTLSGADDFLPILIYITIKANPPQLHSNLKFIQLFRRETRLISEVEYYLTNLISAKMFIMN
VNGHSLSMEESVFQTHMESARLGNHISVASTNSSQGLGTSTPGQNEESGDTEGLFLTL
VDQFVSE (308 aa)

[Helical bundle domain]

MESPTSPASRLDFYDFIGRMRRPAAADLFHSIRSFLASLSQGGEPNAEVDGGRVQTFFA
EMETAIRDHPLWANATNQEIDNALEGLEKYIMTKLFDRAFAS

[Vacuolar Protein Sorting 9 domain]

SAEDVKSDMEISEKIGLLQHFVRPHHLDIPKLLHNEAAWLLAVKELQKINSFKSPREKLSC

IMSCCQVINNLLLNVSMSNDRTLSGADDFLPILIYITIKANPPQLHSNLKFIQLFRRETRLIS
EVEYYLTNLISAKMFIMNVN

Supplement Figure S7. cDNA and the deduced amino acid sequences corresponding to Os03g0842700.

A: cDNA sequence corresponding to Os03g0842700.

B: Deduced amino acid sequences corresponding to Os03g0842700.

Amino acids highlighted with blue and red indicate the helical bundle domain and VPS9 domain.

A

```
CLUSTAL 2.1 multiple sequence alignment


GLUP6/GEF       -MDGGGGGDAFGSATAPLAWHDFLERMRQPSAADFVKSIKGFIVTFSN-RAPDPEHDSAA
Rab5-GEF2       -MESP------TSPASRLDFYDFIGRMRRPAAADLFHSIRSFLASLSQGGEPNAEVDGGR
VPS9a           -MEN---------TDVFLGLHDFLERMRKPSAGDFVKSIKSFIVSFSN-NAPDPEKDCAM
Rabex-5         SINRQTS---IETDRVSKEFIEFLKTF-HKTGQEIYKQTKLFLEGMHYKRDLSIEEQSEC
VPS9b           -MEN---------TDVVSGLHNFLS---KPSAKDFIKSIKSFIVSILN-TAPDPEKDCDA
                 ::                  :*:    : :. :: :. : *:  :      . * :

GLUP6/GEF       VQEFLENMEGAFRAHTPWAGSSEEELESAGEGLEKYVMTKLFNRVFAS
Rab5-GEF2       VQTFFAEMETAIRDHPLWANATNQEIDNALEGLEKYIMTKLFDRAFAS
VPS9a           VQEFFSKMEAAFRAHPLWSGCSEEELDSAGDGLEKYVMTKLFTRVFAS
Rabex-5         AQDFYHNVAERMQTR---GKVPPERVEKIMDQIEKYIMTRLYKYVFCP
VPS9b           VQDFFYKMESAFRAHPLWSGCSDDELDNAGDGLEKYVMTKLFPRVFAS
                .* *  ::   :: :   .  . :.::.  : :***:**:*:  .*..
```

B

Similarity of helical bundle		
Rab5-GEF2	GLUP6/GEF	59.1%
Rab5-GEF2	VPS9a	65.3%
Rab5-GEF2	VPS9b	64.4%
Rab5-GEF2	Rabex-5	40.9%

Supplement Figure S8. Alignment of helical bundle domain from different GEFs

A: Alignment of helical bundle domain in rice (GLUP6/GEF and Rab5-GEF2), *Arabidopsis* (VPS9a and VPS9b), and *Homo sapience* (Rabex-5)

The number of amino acid of the helical bundle of GLUP6/GEF, Rab5-GEF2, VPS9a, Rabex-5, and VPS9b are 106, 101, 97, 101, and, 94, respectively.

B: Similarity of helical bundle domain in Rab5-GEF2 to other proteins.

Supplemental Table S1. List of PCR primers.

Primer	Sequences (5' to 3')
1	CTCGCGAATTCCAACCCCG
2	GATTCTTAGACGAGACAAGAACTCC
3	GGTTCCGCGTGGATCCGATGGCGGCGGCGGAGG
4	GATGCGGCCGCTCGAGTCATGCATGTTCAGGTTGATCTGATG
5	GGTTCCGCGTGGATCC GAGAGCCCCACGTCGCCG
6	GATGCGGCCGCTCGAGTTATTCTGAAACAAACTGATC
7	GGTTCCGCGTGGATCC ACCAAATTGTTCGATCGAG
8	GGTTCCGCGTGGATCCACAAAGCTGTTCAATCGGGTATTTG
9	GATGCGGCCGCTCGAGTCACAGTCCAGGACCTTGGAAATTATC
10	GGTTCCGCGTGGATCCGGTTGCTCCTCCTCCGTG
11	GATGCGGCCGCTCGAGTCAAGACGCCGTTGGCCT
12	GGTTCCGCGTGGATCCGCGGCCAACGCCGGC
13	AGTCACGATGCGGCCGCTCAAGAGCAGCATGAAGCAG
14	GGTTCCGCGTGGATCCGGGGACGAGTCGGAGGG
15	GATGCGGCCGCTCGAGCTAGCAACATCCAAACCGG
16	GGTTCCGCGTGGATCCGCGGCCAACCCCGGCAA
17	GATGCGGCCGCTCGAGTCACTGCATGATCATCCTCTCAGCT

***Rab5-GEF2* full length cDNA clone**

PCR reaction was conducted with the primers 1 and 2 using cDNA reverse-transcribed from mRNA of the immature seeds of the wild type. PCR product was purified and inserted into pUC19 by using in-fusion cloning kit.

GLUP6/GEF expression

PCR reaction was conducted with the primer 3 and 4 using full-length cDNA clone(metioned above) as template. PCR product were purifed and inserted into pGEX4T-1 vector by using In-fusion cloning kit.

Rab5-GEF2 expression

PCR reaction was conducted with the primers 5 and 6 using *Rab5-GEF2* full-length cDNA clone (metioned above) as template. PCR product was purifed and inserted into pGEX4T-1 vector by using In-fusion cloning kit.

Δhelical bandle of Rab5-GEF2 expression

PCR reaction was conducted with the primers 6 and 7 using EST clone (accesion No. AK070821) which encoding Rab5-GEF2 as template. PCR product were purifed and inserted into pGEX4T-1 vector by using In-fusion cloning kit.

Δc-terminal of GLUP6/GEF expression

PCR reaction was conducted with the primers pairs 3 and 9 using GLUP6/GEF full-length cDNA clone (metioned above) as template. PCR products were purifed and inserted into pGEX4T-1 vector by using In-fusion cloning kit.

Δhelical bundle, Δc-terminal of GLUP6/GEF expression

PCR reaction was conducted with the primers pairs 8 and 9using GLUP6/GEF full-length cDNA clone (metioned above) as template. PCR products were purifed and inserted into pGEX4T-1 vector by using In-fusion cloning kit.

Rab GTPase expression

PCR reactions were conducted with the primer pairs, 10 and 11, 12 and 13, 14 and 15, and 16 and 17 using EST clone (accesion No. AK067459, AK 121527, AK103220, AK061116) which encoding, Rab5b, Rab5c, Rab11, GLUP4/Rab5a as template. Then PCR product was purified and inserted into pGEX4T-1 vector by using In-fusion cloning kit.
